# Multi‐Target Mechanisms of Moroccan Aphrodisiac Plants: An Integrative Computational and Phytochemical Investigation

**DOI:** 10.1002/fsn3.71927

**Published:** 2026-05-24

**Authors:** Amal Elrherabi, Oussama Khibech, Mohamed Bouhrim, Abdelouahid Laftouhi, Abdelaaty A. Shahat, Abdullah R. Alanzi, Rashed N. Herqash, Joe Miantezila Basilua, Mohamed Bnouham

**Affiliations:** ^1^ Pharmacology Unit, Laboratory of Bioresources, Biotechnology, Ethnopharmacology, Faculty of Medicine and Pharmacy Mohammed First University Oujda Morocco; ^2^ TBC Laboratories, Department of Pharmacy, UFR3S University of Lille Lille Cedex France; ^3^ Laboratory of Applied Chemistry and Environment, Faculty of Science Mohammed First University Oujda Morocco; ^4^ Biological Engineering Laboratory, Faculty of Sciences and Techniques Sultan Moulay Slimane University Beni Mellal Morocco; ^5^ Laboratory of Electrochemistry, Modeling and Environment Engineering (LIEME), Faculty of Sciences Fes Sidi Mohamed Ben Abdellah University Fes Morocco; ^6^ Department of Pharmacognosy, College of Pharmacy King Saud University Riyadh Saudi Arabia; ^7^ Center for Research on Medicinal, Aromatic, and Poisonous Plants, DSR King Saud University Riyadh Saudi Arabia; ^8^ Department of Biostatistics and Mathematics, Faculty of Pharmacy Paris Cité University Paris France

**Keywords:** aphrodisiac, ethnopharmacology, in silico screening, MM/GBSA, molecular docking, molecular dynamics, moroccan medicinal plants, phosphodiesterase‐5

## Abstract

This integrative study investigates predicted multi‐target mechanisms of Moroccan medicinal plants traditionally used as aphrodisiacs by combining ethnopharmacology, phytochemistry, molecular docking, ADMET filtering, molecular dynamics (MD), and MM/GBSA analyses. A rigorous review identified 33 pharmacologically validated species (20 families) and curated 94 phytochemicals selected according to reported abundance, recurrence in phytochemical studies, and relevance to documented sexual‐health bioactivities. Docking against PDE5, androgen receptor (AR), estrogen receptor‐alpha (ERα), and aromatase highlighted top‐scoring representatives: lutein (PDE5, −10.6 kcal/mol), baicalein (AR, −9.5 kcal/mol), liquiritigenin (ERα, −9.2 kcal/mol), and procyanidins/diosgenin (aromatase, −9.7 kcal/mol). The best‐ranked ligand for each protein was examined to describe interaction fingerprints involving hydrogen‐bond anchoring, hydrophobic packing, and π‐mediated stabilization. Because docking scores alone are insufficient to infer biological activity, the two endocrine hits with favorable docking and acceptable developability profiles, liquiritigenin‐ERα and baicalein‐AR, were further evaluated by 200 ns MD simulations and MM/GBSA calculations using 100 snapshots from the equilibrated 175–200 ns window. These analyses supported time‐dependent complex stability and favorable predicted binding free energies. SwissADME and ADMET‐AI profiling indicated that many candidates occupy a drug‐like physicochemical range, but also flagged important solubility and formulation constraints for highly lipophilic or highly polar chemotypes. Overall, the results provide computational hypotheses for prioritizing Moroccan aphrodisiac phytochemicals; experimental validation through enzyme inhibition assays, receptor/cell‐based studies, and pharmacokinetic/safety testing is required before pharmacological or clinical conclusions can be drawn.

## Graphical abstract

Moroccan medicinal plants with in silico‐approved aphrodisiac effects provide 94 phytochemicals. Docking against PDE5, AR, ERα, and aromatase gives top scores for lutein (−10.6), baicalein (−9.5), liquiritigenin (−9.2), and procyanidins (−9.7). ADMET filtering passes baicalein and liquiritigenin but excludes lutein and procyanidins. MD simulations (200 ns) and MM/GBSA confirm stable baicalein–AR (ΔG ≈ −31) and liquiritigenin–ERα (ΔG ≈ −37) complexes, prioritizing them for experimental validation.

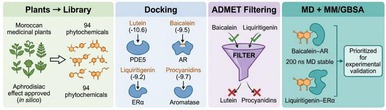

## 1 Introduction

Sexual health and fertility are key dimensions of overall health and wellbeing. Recent global estimates indicate that infertility affects roughly one in six people of reproductive age worldwide (Harris 2023), while large population‐based surveys show that erectile dysfunction (ED) alone affects a substantial proportion of adult men and increases with age (Mark et al. 2024). Sexual health is tightly linked to physical, mental and social wellbeing; disturbances in sexual function are consistently associated with poorer quality of life, relationship distress and psychological burden in both women and men (Vasconcelos et al. 2024). Importantly, erectile dysfunction (ED) and infertility are distinct clinical entities: ED refers to impaired penile erection sufficient for satisfactory sexual performance, whereas infertility refers to the inability to achieve pregnancy after a defined period of regular unprotected intercourse. Accordingly, ED and infertility should not be treated as interchangeable outcomes, although they may coexist and share upstream determinants (e.g., endocrine imbalance, vascular dysfunction, inflammation, and oxidative stress) (Capogrosso et al. 2021; Wang et al. 2023). Thus, sexual dysfunction and infertility represent not only clinical problems but also major public‐health and socio‐economic challenges. From a biological standpoint, sexual disorders often mirror underlying endocrine, vascular, inflammatory and oxidative disturbances that can compromise sexual function and, independently, reproductive capacity. Increasing evidence implicates oxidative stress and inflammation as central mechanisms linking metabolic and infectious insults to impaired gametogenesis, sperm dysfunction and subfertility (Potiris et al. 2025). In men, excessive reactive oxygen species can damage sperm membranes and DNA, disrupt mitochondrial function and reduce fertilization potential, while in women oxidative imbalance affects oocyte quality, implantation and pregnancy maintenance (Liu et al. 2025). Plant‐based antioxidants and other bioactive compounds are consequently attracting attention as adjuncts capable of modulating redox balance, mitochondrial function and inflammatory pathways in the reproductive system (Chorosho et al. 2023).

Conventional pharmacological management of sexual dysfunction includes several target‐directed approaches rather than a single therapeutic class. PDE5 inhibitors such as sildenafil revolutionized the treatment of ED by enhancing nitric oxide‐cGMP‐mediated cavernosal vasodilation (Goldstein et al. 1998), while hormonal therapies, psychosexual interventions, and other pharmacological agents may be used depending on the clinical context; validated tools such as the International Index of Erectile Function (IIEF) are routinely used to quantify therapeutic response (Rosen et al. 1997). Nevertheless, PDE5 inhibitors and other agents (e.g., selective serotonin reuptake inhibitors for premature ejaculation) are not effective or suitable for all patients and may be limited by contraindications, adverse effects, drug–drug interactions, cost, and restricted access, particularly in low‐ and middle‐income settings (Abdelwahab and Taha 2024). These limitations have stimulated renewed interest in complementary strategies that may act on several biological axes while remaining culturally acceptable and more affordable. Across cultures, medicinal plants have long been used as aphrodisiacs to enhance libido, erectile function, fertility, and overall sexual vitality. In the present study, erectile dysfunction and infertility are treated as distinct outcomes: the mechanistic framework centers on sexual‐performance‐associated phytochemicals, particularly PDE5‐mediated ED pathways, while fertility‐related claims are discussed only when supported by independent reproductive evidence (Ganapathy et al. 2021; Ongaro et al. 2021; Yang et al. 2023). Ethnopharmacological and clinical reviews highlight a wide array of plant‐based preparations employed for male and female sexual dysfunction, including tonics, decoctions, powders, and complex polyherbal formulas (Ajao et al. 2019). Contemporary systematic reviews and experimental studies indicate that many of these species contain phytochemicals with documented effects on spermatogenesis, steroidogenesis, endothelial function and sexual behavior in animal models and, to a lesser extent, in humans (Nouioura et al. 2024). Recent work has, for example, demonstrated robust aphrodisiac and testosterone‐enhancing effects of polyherbal formulations based on Apiaceae species traditionally used in North Africa (Nouioura et al. 2024), and catalogued numerous plant‐derived products with potential utility for male sexual dysfunction (Bubnova and Galchenko 2024). At the same time, surveys of “natural aphrodisiacs” underline the heterogeneity of evidence and the importance of better mechanistic and safety data (Shamloul 2010).

These biological activities are largely attributed to diverse classes of secondary metabolites: flavonoids, phenolic acids, saponins, phytosterols, alkaloids, terpenoids, and coumarins that can modulate endocrine and vascular pathways relevant to sexual function (Hmidouche et al. 2023). Many such compounds act as antioxidants and anti‐inflammatory agents, up‐regulate endothelial nitric oxide synthase, influence hypothalamic–pituitary–gonadal signaling, or interact with key enzymes and receptors involved in androgen, estrogen, and cGMP homeostasis (Chorosho et al. 2023). This rich molecular diversity positions medicinal plants as promising sources of multitarget leads for managing sexual dysfunction and related endocrine/vascular disturbances relevant to sexual health, particularly where oxidative and inflammatory processes are prominent (Liu et al. 2025). Morocco is recognized as a Mediterranean biodiversity hotspot and a cradle of sophisticated traditional herbal medicine. Classical repertories and contemporary ethnobotanical surveys document hundreds of medicinal taxa used by Moroccan communities, often with precise indications, preparation methods, and dose regimens (Kachmar et al. 2021). The Moroccan pharmacopoea includes numerous species indicated for “strengthening” the body, treating reproductive disorders, or enhancing sexual performance (Bellakhdar et al. 1991). More recent ethnomedicinal and phytochemical investigations have further characterized emblematic taxa such as *Euphorbia resinifera* and other endemic species with complex bioactive profiles (Hmidouche et al. 2023). In parallel, regional surveys have captured the composition and uses of culturally important mixtures such as Ras El Hanout, a complex spice and medicinal blend widely valued as a tonic and aphrodisiac, as well as local aphrodisiac formulas employed by traditional healers and gynecologists in regions such as Souss Massa (Khalid and Ahmed 2023). These studies demonstrate both the depth of Moroccan ethnopharmacological knowledge and the central place of plant‐based remedies in sexual and reproductive healthcare. Despite this rich ethnopharmacological heritage and the growing body of phytochemical and pharmacological data, important mechanistic gaps remain. Many Moroccan and other traditional aphrodisiac plants have been catalogued with their folk indications and general bioactivities, yet only a minority have been studied in depth for their effects on defined reproductive endpoints such as hormone profiles, sperm parameters, or sexual behavior in rigorous models (Merrouni and Elachouri 2021). Even fewer investigations have systematically linked specific phytoconstituents to molecular targets directly implicated in sexual function, such as the androgen receptor (AR), estrogen receptor alpha (ERα), aromatase, PDE5, or nitric oxide related signaling proteins, or explored how multiple compounds from a single plant or polyherbal mixture might act in concert (Nasim et al. 2022). As a result, the rational prioritization of candidate plants and compounds for further pharmacological or clinical development remains challenging, and the scientific basis for many traditional aphrodisiac practices in Morocco is still fragmentary. Recent advances in computational chemistry and systems pharmacology provide powerful tools to address these gaps. Molecular docking, molecular dynamics, and related *in silico* techniques allow rapid, cost‐effective prediction of binding modes and affinities between natural products and multiple protein targets, thereby helping to uncover plausible mechanisms of action for complex phytochemical mixtures (Trott and Olson 2009). These approaches have already been applied to nutraceuticals (Agu et al. 2023), natural antifungal candidates derived from Moroccan flora (Yamari et al. 2024), and essential oil blends designed to inhibit metabolic enzymes such as carbohydrate‐digesting hydrolases (Loukili et al. 2025). In parallel, the emerging discipline of network pharmacology conceptualizes herbal preparations as multitarget systems and offers frameworks to integrate ethnobotanical knowledge, chemical space, protein–protein interactions, and disease pathways (Hopkins 2008). Together, these tools can bridge traditional knowledge and modern pharmacology by illuminating how individual constituents and mixtures engage with interconnected hormonal and vascular networks that govern sexual function.

Despite the rich Moroccan ethnopharmacological heritage, a coherent mechanistic synthesis linking aphrodisiac use, phytochemical composition, and structure‐based target prediction remains limited. Most Moroccan natural‐product docking studies have focused on infectious or metabolic indications (Haddou et al. 2023), whereas reviews of natural aphrodisiacs rarely integrate compounds from Morocco's flora with defined sexual‐health targets (Bouslamti et al. 2023). Therefore, this study reviews Moroccan medicinal plants documented as aphrodisiacs or sexual tonics, summarizes their major bioactive constituents and pharmacological evidence relevant to sexual and reproductive health, and evaluates the predicted affinity of selected phytochemicals toward PDE5, AR, ERα, aromatase, and related endocrine/vascular nodes using validated docking, ADMET filtering, MD, and MM/GBSA analyses. The aim is not to claim biological efficacy from computation alone, but to generate testable hypotheses and prioritize candidates for future biochemical, cellular, and in vivo validation.

## 2 Materials and Methods

### 2.1 Literature Review and Compilation of Moroccan Aphrodisiac Plants

To establish a scientifically informed database of Moroccan plants with traditional aphrodisiac uses, a systematic review of the ethnobotanical and ethnopharmacological literature was conducted. The process consisted of three sequential phases:

#### 2.1.1 Phase 1: Ethnobotanical Identification and Traditional Use Documentation

A comprehensive search was performed using scientific databases including PubMed, Scopus, Web of Science, and Google Scholar. Keywords and phrases such as “Moroccan medicinal plants,” “aphrodisiac,” “sexual dysfunction,” “ethnobotany Morocco,” “sexual tonic,” “reproductive health Morocco,” and “traditional medicine Morocco” were employed, with no date restrictions. This search aimed to identify all plant species documented within Moroccan traditional medicine systems for the enhancement of libido, erectile function, fertility, or general sexual vitality. Classical Moroccan pharmacopeias and regional ethnobotanical surveys were given particular emphasis (Khalid and Ahmed 2023; Bellakhdar 1997).

#### 2.1.2 Phase 2: Validation of Pharmacological Activity

For each species identified in Phase 1, a secondary literature search was conducted to identify contemporary preclinical (animal) and, where available, clinical (human) studies supporting its aphrodisiac or reproductive‐enhancing effects. Search terms included the plant's scientific name combined with terms like “sperm quality,” “testosterone,” “libido,” “erectile function,” “sexual behavior,” “fertility,” “androgen,” and “estrogen.” Only species with at least one published study in a peer‐reviewed journal demonstrating a significant positive effect on a relevant reproductive or sexual parameter (e.g., increased mating frequency, improved sperm count/motility, elevated serum testosterone or LH levels, enhanced erectile response) were retained for further analysis. This critical filtering step ensured the selection was grounded not only in traditional knowledge but also in modern experimental evidence.

#### 2.1.3 Phase 3: Phytochemical Profiling and Ligand Selection

For the final list of validated species, a detailed review of the phytochemical literature was performed. The term ’principal phytochemicals’ was defined operationally as compounds that were reported as abundant or dominant constituents in chromatographic or spectrometric analyses of the traditionally used plant part, repeatedly reported across independent phytochemical studies, and biologically characteristic of the plant or chemical class associated with the documented sexual‐health activity. Primary and review articles reporting the chemical composition of essential oils, extracts, and isolated compounds were examined. Compounds with unclear identity, unavailable structures, or insufficiently supported occurrence were excluded. This tripartite review strategy—from traditional use to pharmacological validation to chemical characterization—resulted in a final curated list of 94 phytoconstituents representing the major chemical classes implicated in the sexual‐health effects of the selected Moroccan flora.

### 2.2 Molecular Docking

**FIGURE 1:**
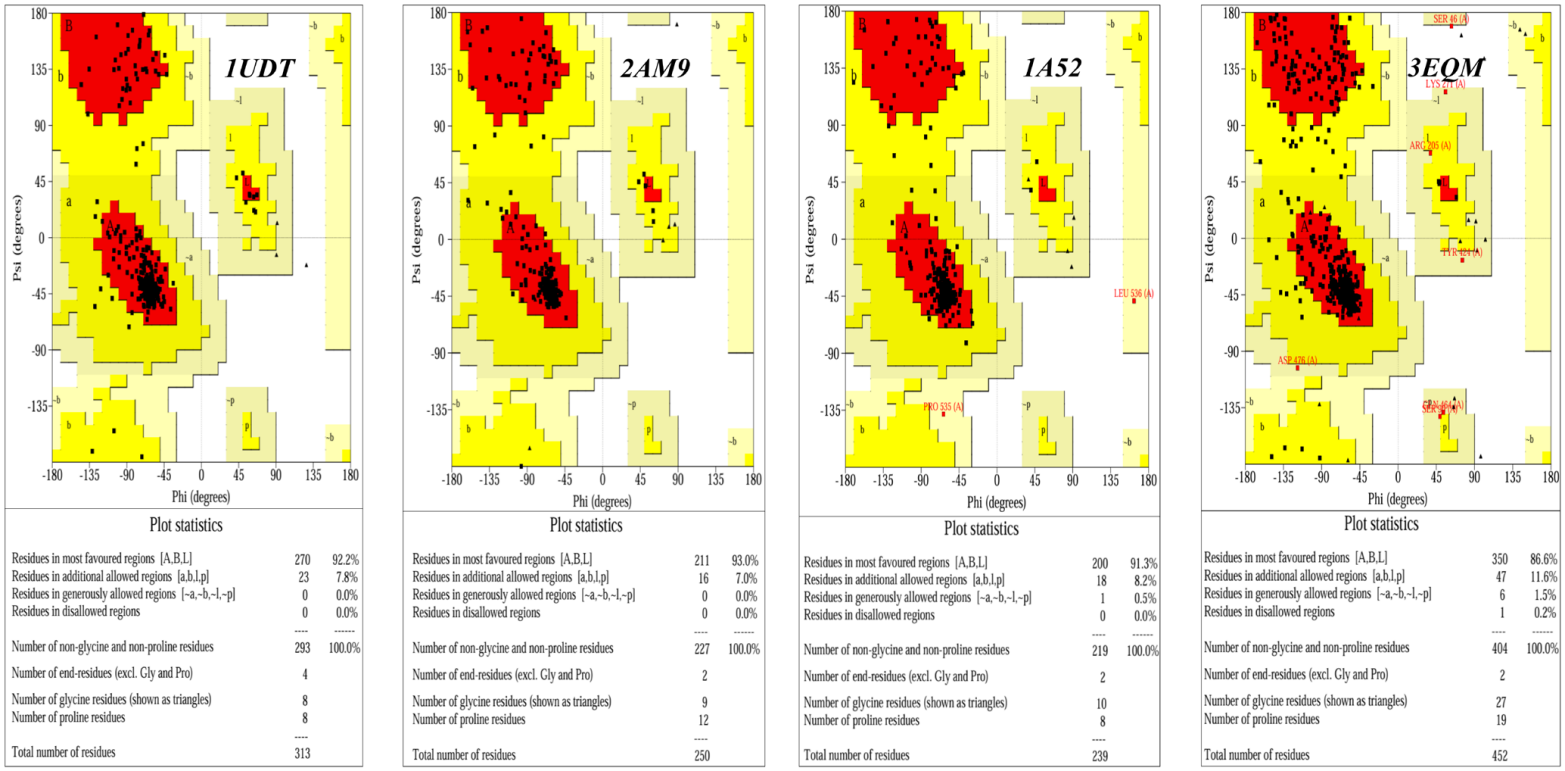
Ramachandran plots for PDE5 (1UDT), AR (2 AM9), ERα (1A52), and aromatase (3EQM) showing the distribution of residues in favored, allowed, and disallowed regions.

The crystal structures of PDE5 (1UDT), AR (2 AM9), ERα (1A52) and aromatase (3EQM) were first evaluated for stereochemical quality by Ramachandran analysis (Figure 1). In all four proteins, more than 86% of non‐glycine/non‐proline residues fell in the most favored regions and almost none in disallowed regions (only a single outlier in 3EQM), confirming that the backbones are suitable for structure‐based modeling. Protein preparation was performed in AutoDockTools 1.5.7: co‐crystallized ligands, crystallographic waters and non‐essential heteroatoms were removed, polar hydrogens were added, non‐polar hydrogens were merged, Kollman‐type charges were assigned, and the receptors were saved in PDBQT format. For each target, the docking grid was centered on the original ligand and adjusted to fully encompass the binding pocket. The grid coverage was visually inspected to ensure inclusion of the co‐crystallized ligand and the key binding‐site amino acids. For 1UDT, the grid centre was set at (1.77, 68.35, 83.21) Å with box dimensions of 11.96 × 18.16 × 14.07 Å; for 2 AM9 at (26.78, 3.29, 4.88) Å with 14.31 × 16.70 × 12.61 Å; for 1A52 at (106.30, 14.49, 96.43) Å with 11.13 × 16.19 × 16.36 Å; and for 3EQM at (85.52, 53.76, 46.03) Å with 13.22 × 14.65 × 12.86 Å. Docking calculations were carried out with AutoDock Vina 1.2.0 as implemented in PyRx 0.8, using an exhaustiveness value of 8 while keeping the remaining parameters at their default settings (Eberhardt et al. 2021).

**FIGURE 2:**
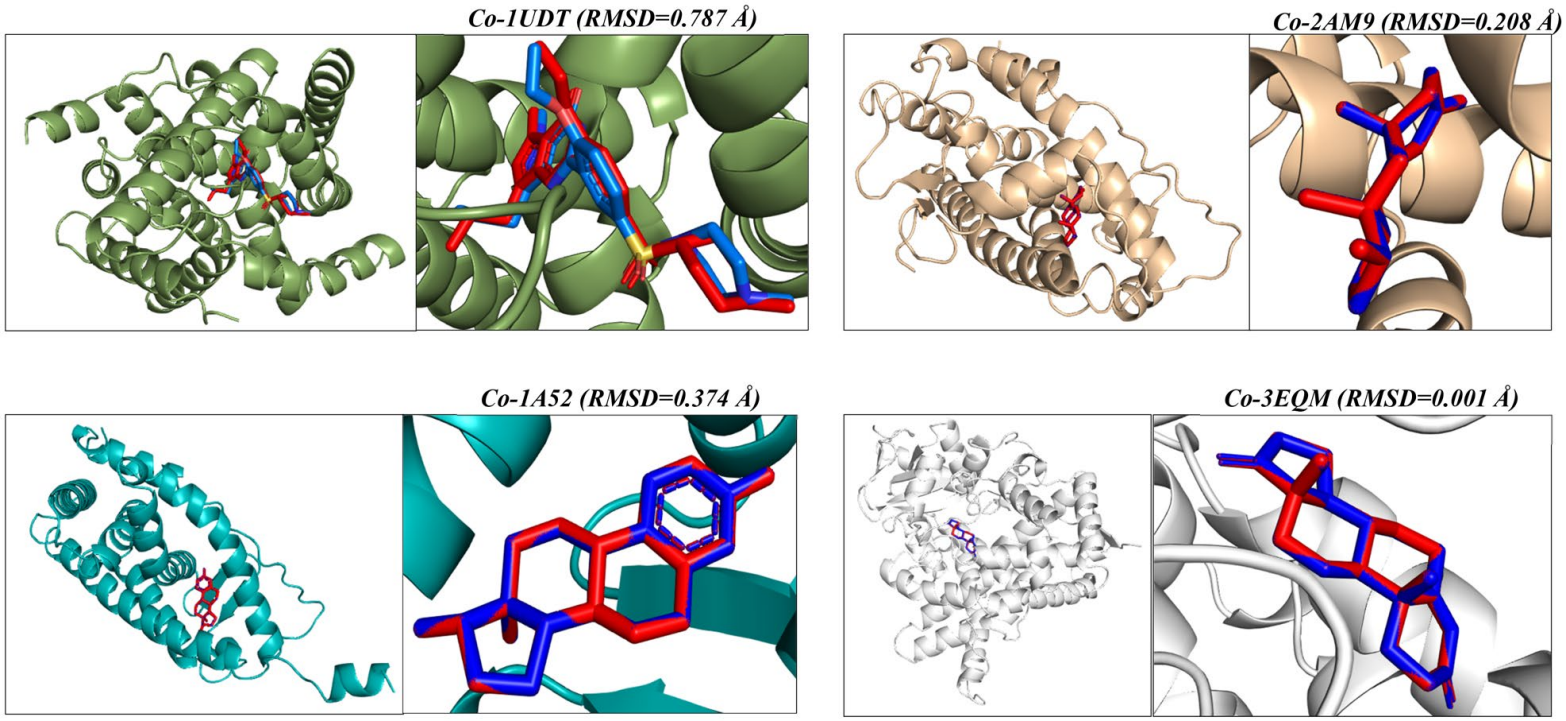
PyMOL‐based validation of the docking protocol by re‐docking the co‐crystallized ligands into PDE5 (1UDT), AR (2 AM9), ERα (1A52), and aromatase (3EQM); experimental poses (blue), and re‐docked poses (red) are overlaid after least‐squares superposition, showing low RMSD values.

The ligand library comprised 94 major phytoconstituents identified in Moroccan medicinal plants. Ligand names were compiled in a plain‐text file and processed by an in‐house Python script, which automatically generated the corresponding SMILES strings and converted them into 3D SDF files. These structures were then imported into PyRx 0.8, where they were protonated according to physiological pH and converted to PDBQT format before docking into each receptor grid. The docking protocol was validated by re‐docking the native co‐crystal ligands of 1UDT, 2 AM9, 1A52, and 3EQM; the close superposition between the crystallographic (blue) and re‐docked (red) poses, with RMSD values ≤ 0.8 Å, confirms that the chosen search space and scoring setup reliably reproduce the experimentally observed binding modes (Figure 2).

### 2.3 In Silico ADMET Prediction Using ADMET‐AI

ADMET properties were predicted in silico using the ADMET‐AI platform, which applies machine‐learning and deep‐learning models to estimate key pharmacokinetic and safety endpoints (absorption, oral bioavailability, toxicity risk, CYP inhibition, clearance, and half‐life) for all screened compounds; the predicted profiles were then used to prioritize the top docked candidates within the DrugBank reference chemical space (Swanson et al. 2024).

### 2.4 Implementation of Molecular Dynamics Simulations Using GROMACS

Molecular dynamics (MD) simulations were performed using GROMACS 2021.3. The AMBER99SB‐ILDN force field was applied to describe the protein, while water molecules were modeled using the TIP3P water model (Merimi et al. 2025; Khibech, Ouachekradi, et al. 2025; Khibech, Bouammali, and Hammouti 2025). Each protein–ligand complex was inserted into a cubic periodic simulation box, maintaining a minimum distance of 1.0 nm between the solute and the box edges. The systems were then solvated with TIP3P water, neutralized by the addition of appropriate counterions, and adjusted to physiological ionic strength with 0.15 M NaCl.

Energy minimization was carried out using the steepest‐descent algorithm for a maximum of 50,000 steps, or until the maximum force reached below 1000 kJ·mol^−1^·nm^−1^. The minimized systems were subsequently equilibrated in two consecutive phases: first under the NVT ensemble at 300 K, followed by NPT equilibration at 1 bar. After equilibration, production MD simulations were conducted for 200 ns using the leap‐frog integration algorithm with a 2 fs time step.

Nonbonded interactions were calculated using the Verlet cutoff scheme, with the neighbor list updated every 20 steps and a cutoff distance of 1.2 nm. Van der Waals interactions were treated using a force‐switching function between 1.0 and 1.2 nm, whereas long‐range electrostatic interactions were handled with the particle mesh Ewald (PME) method, using a Coulomb cutoff of 1.2 nm, a PME interpolation order of 4, and a Fourier grid spacing of 0.16 nm. All hydrogen‐involving bonds were constrained using the LINCS algorithm with standard settings.

Temperature was maintained at 300 K using the V‐rescale thermostat with a coupling constant of 0.1 ps, applied separately to the Protein_LIG and Water_and_ions groups. Pressure was isotropically coupled at 1 bar, using the Berendsen barostat during NPT equilibration and the Parrinello–Rahman barostat during production, with a coupling constant of 2.0 ps and a compressibility of 4.5 × 10^−5^ bar^−1^. The resulting trajectories were analyzed to assess the conformational stability of the complexes and to monitor the persistence and evolution of key protein–ligand interactions throughout the simulations.

### 2.5 MM/GBSA Calculation

Binding free energies (ΔG_bind_) were estimated using the MM/GBSA approach implemented in AmberTools 23 via the MMPBSA.py.MPI module. For each protein–ligand complex, 100 snapshots were uniformly extracted from the equilibrated 175–200 ns interval of the corresponding GROMACS production trajectory.

The polar solvation contribution was calculated using the OBC2 generalized Born implicit‐solvent model (igb = 5), with dielectric constants set to 1.0 for the solute and 80.0 for the solvent, and an ionic strength of 0.15 M. The nonpolar solvation energy was estimated from the solvent‐accessible surface area (SASA).

To further elucidate the molecular determinants of binding, per‐residue energy decomposition was performed using the idecomp = 1 option. This analysis allowed the contribution of individual amino acid residues to the total binding free energy to be quantified and enabled the decomposition of ΔG_bind into its principal energetic components. Temporary and intermediate files generated during the calculations were automatically removed after each run.

## 3 Results and Discussion

### 3.1 Curation of Pharmacologically Validated Plants and Their Phytochemical Profiles

The rigorous three‐phase review strategy successfully transitioned from traditional knowledge to evidence‐based selection. The initial ethnobotanical survey identified numerous species, which were then critically filtered based on the availability of modern experimental data. Table 1 presents the resulting curated list of 33 plant species across 20 families that have demonstrated significant, reproducible effects on sexual and reproductive endpoints in preclinical (animal) and, where available, clinical studies.

**TABLE 1:** Pharmacologically validated effects of major Moroccan medicinal plants on sexual and reproductive health parameters and their principal bioactive constituents.

Family	Species	Medicinal use	Molecules responsible
Zingiberaceae	*Aframomum melegueta*	Aphrodisiac	6‐Paradol, 6‐Gingerol, 6‐Shogaol, gingerols, paradols, a‐Humulene, b‐Caryophyllene, Myrcene, Linalool, [6]‐gingerdione (Khalid and Ahmed 2023; Yu Sheng Toh et al. 2019)
	*Curcuma longa* L.	Improving libido and sexual behavior Increasing testosterone levels Protecting reproductive organs Enhancing sperm count, motility, and viability	Curcumin, Demethoxycurcumin, Turmerone, α‐turmerone, curcumene, germacrone, α‐curcumene, α‐phellandrene (Akhter et al. 2024; Zhang et al. 2024; Kheira et al. 2023)
	*Elettaria cardamomum* (L.) Maton	Aphrodisiac Libido enhancement Sexual vitality Hormonal regulation	α‐Terpinyl acetate, 1,8‐Cineole, Linalool, α‐Terpineol, Linalyl acetate, α‐Pinene (Savan and Küçükbay 2013; Atabaki et al. 2023; Blažeković et al. 2025)
	*Alpinia officinarum*	Enhancing sperm morphology and motility Improving testicular structure Enhancing sexual behavior markers	1,8‐Cineole (Eucalyptol), α‐Fenchyl acetate, Camphene, Geraniol, α‐Terpineol, Linalool, Methyl cinnamate, β‐Pinene, α‐Pinene, Eugenol, δ‐Cadinene (Zhang et al. 2010; Pirzadeh et al. 2021; Negm and Ragheb 2019)
	*Zingiber officinale* Roscoe	Increasing libido Enhancing sexual desire Improving sperm quality Elevating testosterone levels	α‐terpinene, α‐terpineol, 4‐terpineol, terpinolene, gingerols, shogaols, paradols, zingerones, gingerdiones, gingerdiols (Liu et al. 2019; Oktavia et al. 2024; Nihayah et al. 2016; Hadibrata et al. 2025)
Alliaceae	*Allium cepa* L.	Aphrodisiac, emmenagogue	Quercetin‐3,4′‐diglucoside and quercetin‐4′‐monoglucoside, *myricetin*, *quercetin (aglycone)*, *isorhamnetin*. (Fredotović et al. 2017)
	*Allium sativum* L.	Aphrodisiac	Alliin (S‐allyl‐L‐cysteine sulfoxide), Allicin (diallyl thiosulfinate), Ajoene (E‐ and Z‐ajoene), Diallyl disulfide (DADS), Diallyl trisulfide (DATS), S‐allyl cysteine (SAC), S‐allyl mercaptocysteine (SAMC), Vinyldithiins (2‐vinyl‐4H‐1,3‐dithiin, 3‐vinyl‐4H‐1,2‐dithiin), Gallic acid, Caffeic acid, p‐Coumaric acid, Ferulic acid, Vanillic acid, Quercetin, Kaempferol (Talla 2009; Amagase et al. 2001; Lanzotti 2006)
Asteraceae	*Anacyclus pyrethrum* L.	Enhance sexual behavior and reproductive function Increasing sexual activity, motivation, and fertility markers	(2,4)‐N‐isobutyl‐2,4‐undecadiene‐8,10‐diynamide, N‐isobutyl‐dodeca‐2,4,8,10‐tetraenamide, Sarcosine, N‐(trifluoroacetyl)‐butyl ester, Levulinic acid, Propanedioic acid (malonic acid), Palmitic acid, Morphinan‐6‐one, 4,5‐alpha‐epoxy‐3‐hydroxy‐17‐methyl, 2,4 undecadiene‐8,10‐diyne‐N‐tyramide, Isovaleric acid (Talla 2009; Sharma et al. 2009)
	*Saussurea costus* Lipsch	Protecting testosterone and reproductive hormones Improving sperm quality and concentration Increasing ejaculate volume	Chlorogenic acid‐O‐methyl, Dihydroreynosin glucuronide, Costunolide, Dehydrocostus lactone, apigenin, acacetin, baicalein, luteolin, diosmetin; quercetin, kaempferol, isorhamnetin, gossypetin, myricetin (Abd Eldaim et al. 2019; El‐Bolkiny et al. 2025; Barghouth et al. 2025)
Apiaceae	*Apium graveolens* L.	Sexual performances Enhancing reproduction	Socnidilide (cis‐neocnidilide; sedanenolide), β‐Selinene, Senkyunolide A (3‐butyl‐4,5‐dihydrophthalide), Phytyl acetate, 3‐Butylphthalide, Dihydroagarofuran, Kessane, Caryophyllene, Ligustilide (trans‐ligustilide) (Kooti and Asadi‐Samani 2014; Foudah et al. 2021)
	*Coriandrum sativum* L.	Stimulation of spermatogenesis Testicular protection	Linalool, γ‐terpinene, α‐pinene, camphor, neryl acetate, capric acid, 2‐decenoic acid, E‐11‐tetradecenoic acid, geranyl acetate, anethol (Abdul‐Wahaab et al. 2015; Chahal et al. 2017)
	*Daucus carota* L.	Increasing sexual desire Increasing sexual arousal Increasing lubrication Increasing orgasm frequency and satisfaction Increasing receptivity during mating Increasing semen production Enhancing fertility/gestation rate Aphrodisiac activity	Sabinene, α‐pinene, carotol, sabinene (Sadeghi et al. 2020; Bahrami et al. 2018; Aćimović et al. 2016; Momo et al. 2025)
	*Ferula communis* L.	Increasing sex hormones Increasing sexual desire and libido Aphrodisiac potential	Camphor, α‐pinene, β‐eudesmol, α‐eudesmol, δ‐eudesmol, Dillapiole, Spathulenol (Nguir et al. 2016; Macrì et al. 2024; Akaberi et al. 2015)
	*Foeniculum vulgare* Mill.	Inducing estrogen‐like effects Increasing sex hormone levels Improving folliculogenesis	Trans‐anethole, Estragole (methyl chavicol), Fenchone, Limonene (Khazaei et al. 2011; Mallni et al. 1985; Abbas et al. 2020)
	*Petroselinum sativum* Hoffm	Enhancing testosterone Improving sperm quality Protecting reproductive organs	Apigenin, Apiin, Luteolin, Quercetin, Myricetin, Caffeic acid, Chlorogenic acid, Ferulic acid, Apiol, Myristicin, Elemicin, Limonene α‐Pinene, β‐Pinene, 1,8‐Cineole, β‐Carotene, Lutein, β Sitosterol (Slighoua et al. 2023, 2021; Bahramsoltani et al. 2024)
	*Pimpinella anisum* L.	Enhancing libido Improving sexual desire Increasing estrogenic activity Supporting reproductive hormones Reducing menopausal sexual discomfort Improving sexual receptivity	Anethole, Estragole, p‐Methoxyphenylacetone (Weli et al. 2024; Tabanca et al. 2004; Shojaii and Abdollahi Fard 2012)
Asphodelaceae	*Asphodelus microcarpus* Salzm. & Viv.	Traditional aphrodisiac use with no scientific proof.	α‐phellandrene, α‐pinene, Sabinene, β‐Ocimene (Z), δ‐3‐carene (Boumaraf et al. 2024) (Hosni et al. 2020).
Burseraceae	*Boswellia carteri* Birdw	Hormonal modulation	α‐thujene, α‐pinene, α‐phellandrene, 3‐O‐acetyl‐11‐keto‐β‐boswellic acid (AKBA), 11‐keto‐β‐boswellic acid (KBA), β‐Boswellic acid, α‐Boswellic acid, Acetyl‐β‐boswellic acid, Incensole acetate, Octyl acetate (Khalid and Ahmed 2023; Huang et al. 2022; Noktedan et al. 2016)
	*Commiphora myrrha* Engl	Improves sperm parameters Reduces oxidative stress and apoptosis in testes Increases sex hormones	α‐Elemene 12.86, 7‐Isopropyl‐1,4‐dimethyl‐2‐azulenol, Curzerene, Germacra‐1(10),7,11‐trien‐15‐oic acid, 8,12‐epoxy‐6‐hydroxy‐γ‐lactone, δ‐Elemene, δ‐Neoclovene, Germacrene B Eremophilene 3.35% (Mohamed et al. 2014) (Hassanzadeh‐Taheri et al. 2019)
Lauraceae	*Cinnamomum aromaticum* Zoll	Increase sexual performance Increase Ejaculate volume Increased sperm concentration and motility Increased libido	(E)‐Cinnamaldehyde, o‐Methoxy‐cinnamaldehyde, Eugenol, Cinnamyl acetate, Coumarin, α‐thujene, α‐pinene, α‐phellandrene, β‐caryophyllene, δ‐cadinene, α‐copaene, α‐murolene, Flavan‐3‐ols, catechin, epicatechin, procyanidin B‐type oligomers, cinnamic acid (and derivatives), caffeic acid, ferulic acid, p‐coumaric acid, protocatechuic acid, chlorogenic acid, quercetin (and glycosides), kaempferol, myricetin, luteolin, apigenin (Zhang et al. 2019; Goswami et al. 2014; Rao and Gan 2014)
	*Laurus nobilis* L.	Increasing sperm count and motility Enhancing testosterone levels Improving libido Enhancing sexual performance Balancing reproductive hormones	1,8‐cineole, α‐terpinyl acetate, linalool, α‐pinene (Fidan et al. 2019) (Victor et al. 2023) (Falade et al. 2022)
Iridaceae	*Crocus sativus* L.	Improving erectile function Enhancing sexual desire Improving libido Enhancing overall sexual performance	Crocins (cis‐ and trans‐crocins), Picrocrocin, Safranal, Kaempferol (a flavonoid), Trimethyl hydroxy carboxaldehyde cyclohexene (Kashani et al. 2022; Mohammadzadeh‐Moghadam et al. 2015; Shamsa et al. 2009)
Solanaceae	*Datura stramonium* L.	Aphrodisiac activity Increasing semen mass activity, motility, and concentration	Atropine, Hyoscyamine, Scopolamine, 5α‐Ergosta‐7,22‐dien‐3β‐ol, 26,26‐Dimethyl‐5,24 (28)‐ergostadien‐3β‐ol (Zhang et al. 2024; Thawabteh et al. 2025; Owolabi et al. 2024; Mishra and Singh 2009)
Myrtaceae	*Syzygium aromaticum* (L.) Merr. & L.M. Perry	Endocrine Modulation Libido Enhancement Aphrodisiac Activity	Eugenol, Eugenyl acetate, β‐Caryophyllene (Haro‐González et al. 2021; Yilmaz‐Oral et al. 2020; Tajuddin et al. 2004)
	*Myrtus communis* L.	Improving sperm parameters Elevating testosterone levels Protecting reproductive function Supporting fertility	1,8‐Cineole (eucalyptol), α‐Pinene, Linalool, Myrtenyl acetate, Limonene, α‐Terpineol, Myricetin (and derivatives such as myricetin‐3‐O‐arabinoside), Gallic acid, quercetin and other flavonoids (Sumbul et al. 2011; Miraj and Kiani 2016)
Fabaceae	*Glycyrrhiza glabra* L.	Improving libido, and testosterone levels. Aphrodisiac	Glycyrrhizin (Glycyrrhizic acid), 18β‐Glycyrrhetinic acid, Glabridin, Liquiritigenin, Isoliquiritigenin, Liquiritin (Babich et al. 2022; Tanideh et al. 2023)
	*Medicago sativa subsp. sativa* L.	Enhancing female reproductive function Stimulating estrogen and LH secretion Modulating hormonal balance	Chlorogenic acid, Caffeic acid, Gallic acid, Quercetin, Myricetin, Naringenin, Tricin, Medicarpin, Melilotocarpan, Millepurpan, Formononetin, Coumestrol, Beta‐sitosterol and stigmasterol (Krakowska et al. 2017; Al‐Snafi et al. 2021)
	*Trigonella foenum‐ graecum* L.	Improving libido Sexual desire Erectile function in men. Increasing testosterone levels	Diosgenin, Yamogenin, Trigonelline, Isoorientin, Orientin, Vitexin, Isovitexin (Wang et al. 2019; Steels et al. 2011)
Lamiaceae	*Lavandula stoechas* L.	Increasing testosterone levels Improving sperm count and motility and morphology Protecting testicular tissue structure	Fenchone, Camphor, 1,8‐Cineole, Borneol, Linalool, α‐Pinene, β‐Pinene, Sabinene (Sebai et al. 2015) (Elrherabi et al. 2025, 2024; Elrherabi, Bouhrim, Abdnim, et al. 2023; Elrherabi, Bouhrim, Abdnim, Berraaouan, et al. 2023)
	*Rosmarinus officinalis* L.	Supporting testicular function Modulating testosterone levels Regulating reproductive hormones Reproductive organ protection	Carnosic acid, Carnosol, Rosmarinic acid, Ursolic acid, Betulinic acid, 1,8‐Cineole, α‐Pinene, β‐Pinene (Sebai et al. 2015; Andrade et al. 2018)
Brassicaceae	*Lepidium sativum* L.	Improving testosterone, LH, FSH, and Sperm viability and motility in males, Elevating female sex hormones	α‐Linolenic acid, Oleic acid, β‐Sitosterol, γ‐Tocopherol, Quercetin, Kaempferol, Luteolin, Apigenin, Allyl isothiocyanate (Al‐Snafi 2019; Hekmatshoar et al. 2022)
Linaceae	*Linum usitatissimum* L.	Improving semen quality Elevating sex hormone levels	Alpha‐linolenic acid, Secoisolariciresinol diglucoside, Linoleic acid, Oleic acid, quercetin, rutin, catechin hydrate, 4‐hydroxybenzoic acid, β‐Sitosterol (Koçak 2024; Zanwar et al. 2010; Al‐Harbi et al. 2017)
Myristicaceae	*Myristica fragrans*	Enhancing libido Improving erection quality Improving sexual vigor	Sabinene, α‐Pinene, β‐Pinene, β‐Myrcene, Eugenol, Myristicin, Safrole (Warsito 2021; Ahmad et al. 2003)
Ranunculaceae	*Nigella sativa* L.	Enhancing libido Increasing testosterone levels Improving sperm count and motility Elevating LH, FSH, and estrogen Supporting testicular and ovarian structure Boosting fertility and reproductive function Restoring hormonal balance Improving semen quality Promoting ovarian follicle development	Thymoquinone (TQ), Dithymoquinone (DTQ), Trans‐anethole, Carvone, α‐Thujene, Nigellidine, Nigellicine, α‐Hederin (Akram Khan and Afzal 2016; Parandin et al. 2012; Darand et al. 2019)
Cactaceae	*Opuntia ficus‐indica* (L.) Mill	Enhancing testosterone Improving sperm quality Protecting reproductive organs Supporting ovarian follicle development Antioxidant‐mediated fertility support	Oleic acid, Linoleic acid, Palmitic acid, Betalains (indicaxanthin, betanin), Flavonoids (quercetin, isorhamnetin, kaempferol derivatives), Phytosterols (β‐sitosterol) (Abbas et al. 2022; Allai et al. 2023; An et al. 2016)
Nitrariaceae	*Peganum harmala* L.	Enhancing testosterone Improving sperm quality Supporting reproductive organs Modulating ovarian hormones Traditional use as fertility enhancer	Harmine, Harmaline, Vasicine (peganine), α‐Pinene, Sabinene, Limonene (Iranshahy et al. 2019; Derbak et al. 2023; Al‐Mushhadani et al. 2014)
Arecaceae	*Phoenix dactylifera* L.	Enhancing libido Increasing testosterone Improving sperm quality Supporting fertility	Linoleic acid, oleic acid, palmitic acid, β‐sitosterol, stigmasterol, quercetin, luteolin, apigenin, ferulic acid, caffeic acid, p‐coumaric acid, gallic acid, β‐carotene, Lutein (Bahmanpour et al. 2006)
Piperaceae	*Piper nigrum* L.	Enhancing libido Improving sexual behavior Increasing testosterone Improving sperm quality	α‐Pinene, β‐Pinene, Limonene, Linalool, δ‐Elemene, γ‐Elemene, Curzerene, β‐Caryophyllene (Feitosa et al. 2024; Kanedi 2015)
Lythraceae	*Punica granatum* L.	Enhancing libido Improving sexual performance Increasing testosterone Improving sperm quality Supporting erectile function	Punicalagins, Ellagic acid, Gallagic acid, Cyanidin, Delphinidin, Pelargonidin, Catechins, Epicatechins, Quercetin, Kaempferol, Punicic acid, Alkaloids, Vitamin C, Potassium, Saponins, Sterols (Mohammad and Kashani 2012; Leiva et al. 2011)
Rosaceae	*Rosa damascena* Mill.	Enhancing libido Improving sexual behavior Increasing testosterone Aphrodisiac effect	Phenylethyl alcohol, Nerol, Citronellol, Geraniol, 1‐Nonadecene, Nonadecane (Farnia et al. 2017; Verešová et al. 2024)
Urticaceae	*Urtica dioica* L.	Increasing libido Enhancing sexual desire Improving testosterone levels Improving sperm quality Fertility support	5‐O‐Caffeoylquinic acid, Rutin, Isoquercitrin, Kaempferol‐3‐O‐glucoside, Secoisolariciresinol, 9,9′‐Bisacetyl‐neo‐olivil (Francišković et al. 2017; Pourahmadi et al. 2013)
Verbenaceae	*Vitex agnus‐castus* L.	Modulating prolactin Improving sexual desire in women Reducing PMS symptoms Hormonal balance support Enhancing reproductive health	Oleic acid, Linoleic acid, β‐Sitosterol, Stigmasterol (Asdadi et al. 2014; Heirati et al. 2021)
Rhamnaceae	*Ziziphus jujuba* Mill.	Improving testosterone levels Fertility support	Triterpenic acids, Flavonoid derivatives, (−)‐Catechin, Quercetin‐3‐O‐robinobioside, Rutin, Quercetin‐3‐O‐α‐L‐arabinosyl‐(1 → 2)‐α‐L‐rhamnoside, Gallic acid, Chlorogenic acid, Luteolin (Mohebbati et al. 2021; Asgharzadeh et al. 2025)
	*Ziziphus spina‐christi*	Increasing testosterone levels Enhancing luteinizing hormone (LH) Improving follicle‐stimulating hormone (FSH) Enhancing sperm quality Increasing semen parameters Protecting against oxidative stress in reproductive tissues Supporting testicular function	Kaempferol‐3‐O‐rhamnoside, Myricetin‐3‐O‐(6‐rhamnosyl)‐hexoside, Quercetin‐3‐O‐[(2‐hexonyl)‐6‐rhamnosyl]‐hexoside, Quercetin‐3‐O‐p‐coumaroyl‐(2,6‐dirhamnosyl)‐hexoside, nummularine‐E, nummularine‐D, spinanine‐B, spinanine‐C, amphibine‐D, spinanine‐A (Althaher et al. 2024; Abd El Hady et al. 2025)
Zygophyllaceae	*Tribulus terrestris* Linn.	Enhances sexual behavior Improves erectile function Increases testosterone levels	Protodioscin, Protogracillin, Dioscin, Diosgenin, Tribulosin, Tribulosaponins A–E, Quercetin, Kaempferol, Rutin, Isorhamnetin, Harmine, Harmane, Harmaline, β‐Sitosterol, Stigmasterol, Campesterol, N‐trans‐feruloyltyramine, N‐p‐coumaroyltyramine (Semerdjieva and Zheljazkov 2019) (Singh et al. 2012)
Rutaceae	*Ruta chalepensis* L.	Increasing testosterone levels Enhancing LH and FSH Stimulation of sex organs Improving spermatogenesis Enhancing sexual function Androgenic effect Reproductive stimulant	Rutine, Skimmianine, Graveoline, Chalepensin, Quercetin, Rutin, Kaempferol, 2‐Undecanone, 1‐Nonanol, 1‐Decanol, Linalool, α‐Pinene, β‐Pinene, Limonene, Xanthotoxin, Bergapten, Psoralen, Chalepin (Althaher et al. 2024; Al‐Qarawi 2005)

The “Medicinal Use” column details these documented pharmacological activities, which served as the primary criterion for inclusion. These effects include, but are not limited to: enhanced libido and sexual behavior markers, increased serum testosterone and other sex hormone levels, improved sperm parameters (count, motility, morphology), and protective effects on reproductive organ structure. This evidence‐based filter ensures that the subsequent molecular docking analysis focuses on plants with a scientifically substantiated bioactivity profile, moving beyond anecdotal traditional use.

Concurrently, the phytochemical profiling phase (Phase 3) identified the major secondary metabolites for each species. The resulting chemical library, as shown in the “Molecules Responsible” column, is dominated by key classes known for modulating endocrine, vascular, and oxidative pathways: flavonoids (e.g., apigenin, quercetin), phenolic acids, terpenoids (e.g., monoterpenes, sesquiterpenes), alkaloids (e.g., harmine), and steroidal saponins/phytosterols (e.g., diosgenin, β‐sitosterol). This compilation directly links the observed in vivo effects to specific pools of bioactive compounds, providing a solid chemical basis for the *in silico* investigation. Thus, Table 1 serves as the crucial bridge connecting traditional aphrodisiac plants of Morocco to their experimentally validated effects and constituent chemistry, setting the stage for the mechanistic exploration via molecular docking.

### 3.2 Molecular Docking

Molecular docking was used to generate structure‐based hypotheses on the interaction of major Moroccan aphrodisiac phytochemicals with key vascular and endocrine targets. The 94‐compound library was docked against PDE5 (1UDT), AR (2 AM9), ERα (1A52), and aromatase (3EQM), which represent complementary nodes involved in cGMP‐mediated vasodilation and androgen/estrogen signaling. Docking scores were interpreted as predicted binding tendencies rather than experimental evidence of activity. For comparative analysis, compounds with binding energies ≤ −9.0 kcal/mol were considered high‐priority docking hits, those between −7.0 and −8.9 kcal/mol as moderate predicted binders, and those > −7.0 kcal/mol as weak‐to‐low‐priority binders (Kobayashi et al. 2023; Al‐zobaidy et al. 2024; Gao et al. 2016; De Filippis et al. 2025).

As summarized in Table 2, simple volatile terpenes and small phenylpropanoids generally showed weak‐to‐moderate predicted binding, whereas selected flavonoids, condensed tannins, carotenoid/steroidal scaffolds, and lignans reached the high‐priority range for one or more targets. The strongest target‐specific candidates were lutein for PDE5 (−10.6 kcal/mol), baicalein for AR (−9.5 kcal/mol), liquiritigenin for ERα (−9.2 kcal/mol), and procyanidins/diosgenin for aromatase (−9.7 kcal/mol). Overall, bulky hydrophobic or steroid‐like structures showed preferential complementarity with PDE5 and aromatase pockets, while planar flavonoid aglycones were more favorable for AR and ERα ligand‐binding domains (Merimi et al. 2025; Khibech, Ouachekradi, et al. 2025; Rahimi et al. 2025).

**TABLE 2:** Docking scores of the main phytoconstituents from Moroccan medicinal plants against PDE5 (1UDT), androgen receptor (2 AM9), estrogen receptor‐α (1A52), and aromatase (3EQM), highlighting the best‐scoring ligands for each protein.

No	Compound/PDB	1UDT	2 AM9	1A52	3EQM
1	6‐Paradol	−7.3	−7.2	−7.0	−6.2
2	6‐Gingerol	−7.5	−7.9	−7.3	−6.1
3	6‐Shogaol	−7.5	−7.5	−7.3	−6.8
4	Myrcene	−5.6	−5.7	−5.4	−4.9
5	Linalool	−6.0	−6.0	−5.6	−5.1
6	[6]‐Gingerdione	−7.2	−7.3	−7.3	−6.6
7	Turmerone	−7.6	−7.6	−7.7	−6.6
8	Curcumene	−7.9	−7.4	−7.4	−6.6
9	Germacrone	−8.2	−7.5	−7.6	−7.3
10	1,8‐Cineole	−5.4	−6.5	−5.7	−5.3
11	Linalyl acetate	−6.3	−6.5	−6.3	−5.6
12	Camphene	−5.6	−6.5	−5.6	−5.5
13	Geraniol	−6.1	−6.1	−5.8	−5.7
14	Methyl cinnamate	−6.6	−6.6	−6.4	−6.1
15	Terpinolene	−6.6	−6.5	−6.1	−5.7
16	Sabinene	−6.0	−6.0	−5.9	−5.3
17	Incensole acetate	−10.1	−2.8	−7.0	−9.0
18	Octyl acetate	−5.5	−5.7	−4.9	−4.8
19	7‐Isopropyl‐1,4‐dimethyl‐2‐azulenol	−8.8	−7.9	−8.5	−7.5
20	Curzerene	−7.4	−7.6	−7.6	−6.8
21	Germacrene B	−8.7	−6.4	−8.1	−7.4
22	Eremophilene	−8.4	−8.4	−8.2	−7.6
23	Phytyl acetate	−7.6	−6.5	−7.4	−6.5
24	3‐Butylphthalide	−7.4	−7.2	−6.9	−6.2
25	Dihydroagarofuran	−6.9	−8.4	−8.5	−7.2
26	Kessane	−7.5	−8.5	−8.2	−7.3
27	Trans‐anethole	−6.1	−6.2	−5.7	−5.4
28	Estragole	−5.8	−6.1	−5.4	−5.3
29	Fenchone	−5.8	−6.7	−5.8	−5.5
30	Limonene	−6.2	−6.3	−6.0	−5.6
31	Myristicin	−6.6	−6.9	−6.2	−6.3
32	Safrole	−6.6	−6.6	−5.9	−5.7
33	Anethole	−6.1	−6.2	−5.7	−5.4
34	Myricetin	−8.8	−9.0	−7.9	−8.3
35	Isorhamnetin	−8.7	−8.0	−7.8	−7.8
36	Apigenin	−9.3	−9.3	−8.8	−7.7
37	Acacetin	−9.2	−8.0	−7.3	−7.8
38	Baicalein	−8.9	−9.5	−8.7	−8.5
39	Luteolin	−9.3	−9.3	−8.6	−8.0
40	Diosmetin	−9.0	−7.8	−7.1	−7.8
41	Gossypetin	−9.4	−9.1	−8.5	−8.0
42	Kaempferol	−8.9	−9.0	−8.5	−7.8
43	Rutin	−9.7	5.7	−3.7	−9.3
44	Isoquercitrin	−10.0	−2.8	−7.0	−8.7
45	Secoisolariciresinol	−8.2	−6.7	−8.0	−7.5
46	Catechin	−8.5	−9.2	−8.0	−7.8
47	Epicatechin	−8.5	−9.2	−8.0	−7.8
48	Procyanidins	−9.5	−5.6	−5.3	−9.7
49	Picrocrocin	−8.1	−7.9	−7.8	−7.2
50	Safranal	−6.3	−6.7	−6.2	−5.5
51	Gallic acid	−6.0	−6.3	−5.8	−5.8
52	Caffeic acid	−6.7	−7.0	−6.5	−6.2
53	p‐Coumaric acid	−6.8	−6.9	−6.3	−5.9
54	Ferulic acid	−6.7	−6.9	−6.4	−5.9
55	Chlorogenic acid	−8.7	−6.8	−8.3	−7.8
56	Protocatechuic acid	−6.2	−6.3	−5.9	−5.6
57	Coumarin	−7.0	−6.8	−6.3	−6.1
58	Allicin	−4.2	−4.5	−4.4	−3.8
59	Harmine	−8.0	−8.0	−7.4	−7.0
60	Harmane	−7.8	−7.7	−7.6	−6.8
61	Harmaline	−8.0	−7.9	−7.4	−7.0
62	Vasicine (Peganine)	−7.5	−7.5	−7.3	−6.4
63	N‐trans‐feruloyltyramine	−9.0	−6.6	−8.0	−7.7
64	N‐p‐coumaroyltyramine	−8.8	−7.4	−8.7	−7.7
65	Diosgenin	−8.7	−5.3	−7.9	−9.7
66	Glabridin	−10.5	−3.2	−5.8	−9.0
67	Liquiritigenin	−9.1	−9.1	−9.2	−7.6
68	Isoliquiritigenin	−8.4	−8.7	−8.6	−7.4
69	Liquiritin	−9.9	−4.0	−3.3	−8.4
70	Beta‐sitosterol	−10.3	−2.6	−4.1	−9.1
71	Stigmasterol	−10.0	−0.8	−5.7	−9.4
72	Campesterol	−9.7	−3.7	−4.8	−8.9
73	Vitexin	−9.4	−5.8	−6.5	−8.8
74	Isovitexin	−9.0	−6.7	−1.7	−6.9
75	Secoisolariciresinol diglucoside	−9.6	−4.8	−3.3	−8.2
76	Levulinic acid	−4.7	−5.1	−4.5	−4.1
77	Palmitic acid	−6.4	−6.5	−6.2	−5.3
78	Oleic acid	−7.0	−6.5	−6.6	−5.5
79	Linoleic acid	−7.1	−6.8	−6.9	−5.8
80	Capric acid	−5.8	−6.0	−5.3	−4.9
81	2‐Decenoic acid	−5.9	−6.0	−5.6	−5.3
82	E‐11‐Tetradecenoic acid	−6.5	−6.3	−6.0	−5.3
83	Senkyunolide A	−6.7	−7.2	−7.0	−6.1
84	Elemicin	−6.3	−5.8	−6.0	−5.9
85	Apiol	−6.5	−6.4	−6.2	−6.4
86	Dillapiole	−6.5	−6.7	−6.2	−5.8
87	Spathulenol	−7.7	−8.1	−8.0	−7.5
88	Lutein	−10.6	−4.8	−5.3	−6.5
89	Atropine	−7.8	−8.4	−7.5	−7.4
90	Hyoscyamine	−7.8	−8.3	−7.6	−7.4
91	Scopolamine	−8.1	−8.5	−7.9	−7.8
92	Isovaleric acid	−4.6	−5.1	−4.5	−4.1
93	1‐Nonadecene	−6.4	−6.2	−6.0	−5.2
94	Nonadecane	−6.1	−6.2	−6.0	−5.2
	Co‐crystal ligand	−10.1	−11.4	−10.6	−10.7

**FIGURE 3:**
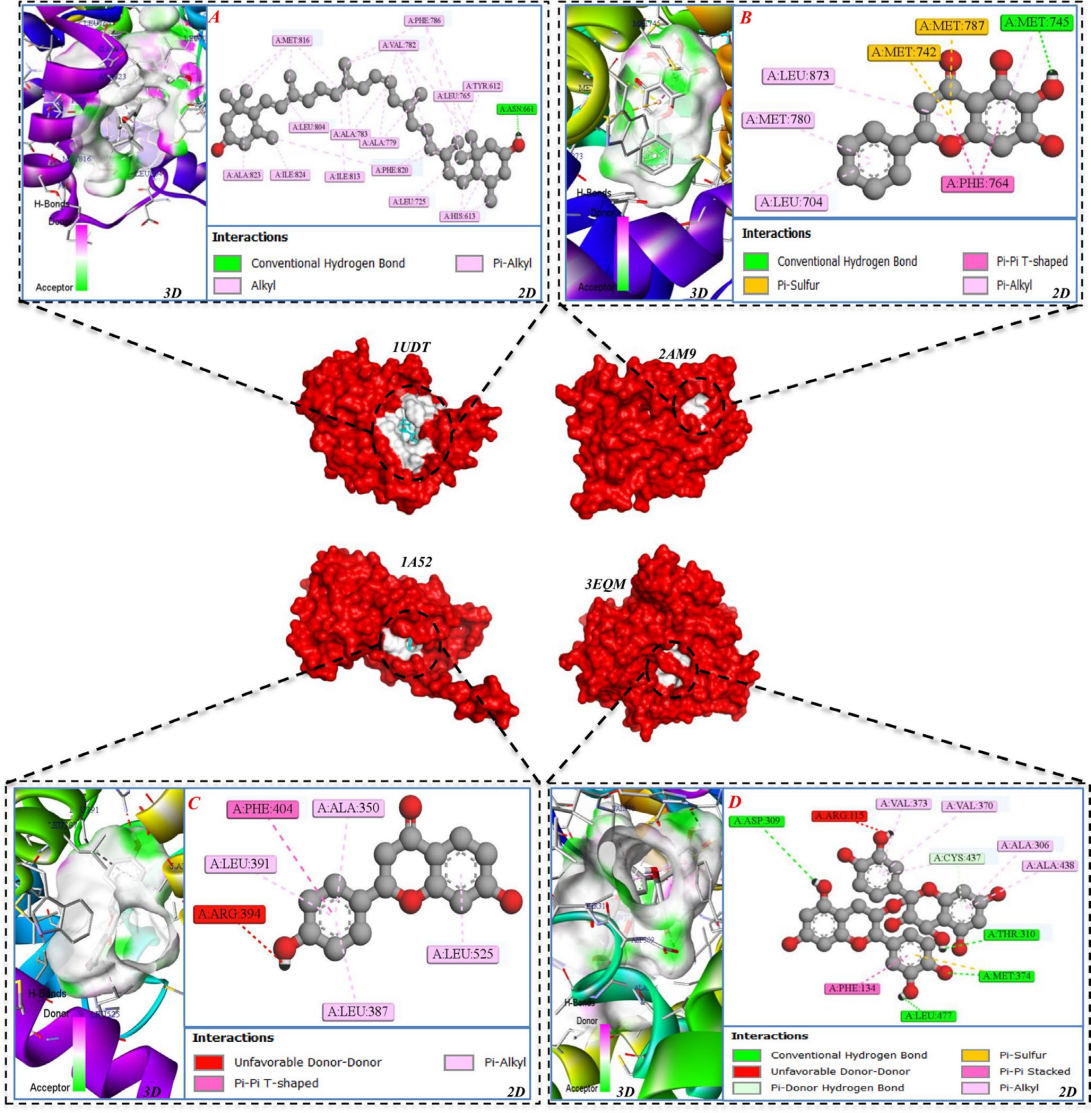
Visualization of ligand binding within the four hormone‐related targets. Proteins are shown as red surface representations, while the binding cavities are highlighted in gray. Panels (A–D) illustrate the 3D binding environment and 2D interaction profiles of lutein (PDE5), baicalein (AR), liquiritigenin (ERα), and procyanidins (aromatase).

Figure 3 illustrates, for each target, the detailed interaction pattern formed by the best‐scoring major constituent, thereby rationalizing why these ligands were selected as representative binders. Lutein (Panel A), baicalein (Panel B), liquiritigenin (Panel C), and procyanidins (Panel D) are not only among the most abundant metabolites in the investigated plants, but also capture four distinct chemotypes: xanthophyll carotenoid, flavone, flavanone, and condensed tannin through which Moroccan phytotherapy may modulate PDE5, AR, ERα, and aromatase at the molecular level. In general, hydrogen‐bond anchoring involves short contact distances (~2.0–3.2 Å), while hydrophobic/π interactions typically occur at longer distances (~3.5–5.5 Å), reflecting packing stabilization rather than directional bonding (Abbaoui, Khibech, Oulous, et al. 2025; Et‐tazy et al. 2025).

In Panel A (1UDT–lutein), the carotenoid chain is anchored by a single conventional hydrogen bond with Asn661, while the elongated, highly conjugated skeleton is surrounded by a dense mesh of alkyl and π–alkyl contacts distributed along both polyene and terminal rings. This predominance of hydrophobic packing, with several dozen van der Waals contacts, is fully consistent with the largely apolar nature of the PDE5 cavity: in the 3D interaction surface, only small patches of donor (mauve) and acceptor (green) regions are visible around the anchoring pole, whereas the pocket is mostly neutral (white) (Abbaoui, Khibech, Oulous, Karci, and Dündar 2025; Hammouti et al. 2025; Ouakil et al. 2025). The complex is therefore stabilized less by discrete polar “anchors” than by extensive shape complementarity and dispersion forces, which explains why a bulky, lipophilic pigment such as lutein emerges as the preferred binder for PDE5.

In Panel B (2 AM9–baicalein), the flavone scaffold engages in a more polar, directionally defined network. A conventional hydrogen bond with Met745, together with two π–sulfur contacts involving Met787 and Met742, provides specific anchoring points within the androgen receptor ligand‐binding domain, complemented by several additional hydrophobic interactions that clamp the aromatic rings in place (Laaraj et al. 2022; Katsoulaki et al. 2025; Benabbou et al. 2025; Hmamou et al. 2026). The 3D surface analysis of the AR–baicalein complex shows mainly hydrogen‐bond acceptor regions around the ligand, indicating that AR residues likely donate H‐bonds to baicalein's carbonyl and phenolic groups. Together with π–sulfur contacts and hydrophobic packing, this interaction pattern explains baicalein's strong docking score and suggests that planar polyphenols may partially mimic androgen recognition.

In the ERα–liquiritigenin complex, binding is mainly supported by hydrophobic contacts with the aromatic core, while the polar interaction network is weaker than that of estradiol. The presence of an unfavorable donor–donor contact with Arg394 suggests suboptimal polar complementarity, consistent with liquiritigenin acting as a moderate predicted ERα binder rather than a full estradiol‐like ligand.

For aromatase–procyanidins, the interaction pattern is more extensive, involving several hydrogen bonds with Asp309, Thr310, Met374, Leu477, and Cys437, reinforced by hydrophobic and π‐mediated contacts. Although an unfavorable contact with Arg115 is observed, the dense favorable interaction network likely explains the strong predicted affinity for aromatase. Overall, Figure 3 shows that each ligand uses a target‐specific balance of hydrophobic, hydrogen‐bonding, and π‐mediated interactions, supporting their prioritization for further validation.

### 3.3 ADMET Results

ADMET profiling was used as a complementary prioritization step because favorable docking does not necessarily translate into oral exposure, metabolic stability, or safety. SwissADME descriptors (Table 3) showed that 70/94 compounds had no Lipinski or Veber violations; however, these rules are necessary screening heuristics and are not sufficient to establish bioavailability. Several top docked compounds, including lutein, β‐sitosterol, stigmasterol, diosgenin, and procyanidins, present substantial developability concerns such as very low predicted solubility (logS < −6, and in some cases much lower), high lipophilicity, high molecular weight, or excessive polarity. Consequently, these molecules may require formulation strategies, structural optimization, or non‐oral delivery considerations before any translational claim can be made (Khibech, Bouammali, and Hammouti 2025; Baddaoui et al. 2026; Tlemcani et al. 2025; Tina et al. 2026; Bouammali et al. 2025; Kadda et al. 2026).

**TABLE 3:** SwissADME drug‐likeness and developability descriptors for the 94 phytochemicals: Molecular weight (M_W_, g·mol^−1^), hydrogen‐bond acceptors (HBA, count), hydrogen‐bond donors (HBD, count), lipophilicity (cLogP, unitless), topological polar surface area (TPSA, Å^2^), rotatable bonds (RotB, count), bioavailability score (unitless), predicted aqueous solubility (logS, log10 mol·L^−1^), Lipinski violations (count), Veber violations (count).

ID	Compound	M_W_	HBA	HBD	cLogP	TPSA	RotB	Bioavail.	LogS	Lipinski viol.	Veber viol.
1	6‐Paradol	278.39	3	1	4.26	46.53	10	0.55	−8.50	0	0
2	6‐Gingerol	294.39	4	2	3.23	66.76	10	0.55	−7.12	0	0
3	6‐Shogaol	276.38	3	1	4.04	46.53	9	0.55	−8.19	0	0
4	Myrcene	136.24	0	0	3.48	0.00	4	0.55	−6.21	0	0
5	Linalool	154.25	1	1	2.67	20.23	4	0.55	−5.19	0	0
6	[6]‐Gingerdione	292.38	4	1	3.44	63.60	10	0.55	−7.41	0	0
7	Turmerone	218.34	1	0	4.21	17.07	4	0.55	−8.15	0	0
8	Curcumene	202.34	0	0	4.84	0.00	4	0.55	−8.91	0	0
9	Germacrone	218.34	1	0	4.36	17.07	0	0.55	−8.56	0	0
10	1,8‐Cineole	154.25	1	0	2.74	9.23	0	0.55	−5.50	0	0
11	Linalyl acetate	196.29	2	0	3.24	26.30	5	0.55	−6.41	0	0
12	Camphene	136.24	0	0	3.00	0.00	0	0.55	−5.70	0	0
13	Geraniol	154.25	1	1	2.67	20.23	4	0.55	−5.19	0	0
14	Methyl cinnamate	162.19	2	0	1.87	26.30	2	0.55	−4.14	0	0
15	Terpinolene	136.24	0	0	3.45	0.00	0	0.55	−6.38	0	0
16	Sabinene	136.24	0	0	3.00	0.00	1	0.55	−5.65	0	0
17	Incensole acetate	348.53	3	0	5.74	35.53	2	0.55	−11.83	1	0
18	Octyl acetate	172.27	2	0	2.91	26.30	7	0.55	−5.58	0	0
19	7‐Isopropyl‐1,4‐dimethyl‐2‐azulenol	214.31	1	1	4.24	20.23	1	0.55	−8.25	0	0
20	Curzerene	218.34	1	0	4.30	13.14	2	0.55	−8.35	0	0
21	Germacrene B	204.36	0	0	5.18	0.00	0	0.55	−9.65	1	0
22	Eremophilene	204.36	0	0	4.73	0.00	1	0.55	−8.92	0	0
23	Phytyl acetate	338.58	2	0	6.93	26.30	14	0.17	−12.93	1	1
24	3‐Butylphthalide	190.24	2	0	3.09	26.30	3	0.55	−6.20	0	0
25	Dihydroagarofuran	222.37	1	0	4.16	9.23	0	0.55	−8.30	0	0
26	Kessane	222.37	1	0	4.02	9.23	0	0.55	−8.09	0	0
27	Trans‐anethole	148.20	1	0	2.73	9.23	2	0.55	−5.28	0	0
28	Estragole	148.20	1	0	2.42	9.23	3	0.55	−4.77	0	0
29	Fenchone	152.24	1	0	2.40	17.07	0	0.55	−4.96	0	0
30	Limonene	136.24	0	0	3.31	0.00	1	0.55	−6.12	0	0
31	Myristicin	192.21	3	0	2.15	27.69	3	0.55	−4.82	0	0
32	Safrole	162.19	2	0	2.14	18.46	2	0.55	−4.55	0	0
33	Anethole	148.20	1	0	2.73	9.23	2	0.55	−5.28	0	0
34	Myricetin	318.24	8	6	1.69	151.59	1	0.17	−5.47	1	1
35	Isorhamnetin	316.26	7	4	2.29	120.36	2	0.55	−6.30	0	0
36	Apigenin	270.24	5	3	2.58	90.90	1	0.55	−6.31	0	0
37	Acacetin	284.27	5	2	2.88	79.90	2	0.55	−6.86	0	0
38	Baicalein	270.24	5	3	2.58	90.90	1	0.55	−6.31	0	0
39	Luteolin	286.24	6	4	2.28	111.13	1	0.55	−6.03	0	0
40	Diosmetin	300.27	6	3	2.59	100.13	2	0.55	−6.58	0	0
41	Gossypetin	318.24	8	6	1.69	151.59	1	0.17	−5.47	1	1
42	Kaempferol	286.24	6	4	2.28	111.13	1	0.55	−6.03	0	0
43	Rutin	610.52	16	10	−1.69	269.43	6	0.17	−3.09	3	1
44	Isoquercitrin	464.38	12	8	−0.54	210.51	5	0.17	−3.40	2	1
45	Secoisolariciresinol	362.42	6	4	2.12	99.38	9	0.55	−6.16	0	0
46	Catechin	290.27	6	5	1.55	110.38	1	0.55	−4.98	0	0
47	Epicatechin	290.27	6	5	1.55	110.38	1	0.55	−4.98	0	0
48	Procyanidins	594.53	13	10	2.73	229.99	4	0.17	−9.65	3	1
49	Picrocrocin	330.38	7	4	−0.49	116.45	4	0.55	−2.20	0	0
50	Safranal	150.22	1	0	2.49	17.07	1	0.55	−5.02	0	0
51	Gallic acid	170.12	4	4	0.50	97.99	1	0.55	−2.21	0	0
52	Caffeic acid	180.16	3	3	1.20	77.76	2	0.55	−3.31	0	0
53	p‐Coumaric acid	164.16	2	2	1.49	57.53	2	0.55	−3.59	0	0
54	Ferulic acid	194.19	3	2	1.50	66.76	3	0.55	−3.85	0	0
55	Chlorogenic acid	354.31	8	6	−0.65	164.75	4	0.17	−2.20	1	1
56	Protocatechuic acid	154.12	3	3	0.80	77.76	1	0.55	−2.49	0	0
57	Coumarin	146.14	2	0	1.79	30.21	0	0.55	−3.94	0	0
58	Allicin	162.28	2	0	1.76	17.07	5	0.55	−3.85	0	0
59	Harmine	212.25	2	1	3.03	37.91	1	0.55	−6.41	0	0
60	Harmane	182.23	1	1	3.02	28.68	0	0.55	−6.14	0	0
61	Harmaline	214.27	2	1	2.54	37.38	1	0.55	−5.71	0	0
62	Vasicine (Peganine)	188.23	3	1	1.30	35.83	0	0.55	−3.64	0	0
63	N‐trans‐feruloyltyramine	313.35	4	3	2.48	78.79	6	0.55	−6.36	0	0
64	N‐p‐coumaroyltyramine	283.33	3	3	2.47	69.56	5	0.55	−6.09	0	0
65	Diosgenin	414.63	3	1	5.71	38.69	0	0.55	−12.56	1	0
66	Glabridin	324.38	4	2	4.00	58.92	1	0.55	−9.00	0	0
67	Liquiritigenin	256.26	4	2	2.80	66.76	1	0.55	−6.52	0	0
68	Isoliquiritigenin	256.26	4	3	2.70	77.76	3	0.55	−6.26	0	0
69	Liquiritin	418.40	9	5	0.28	145.91	4	0.17	−4.22	0	1
70	Beta‐sitosterol	414.72	1	1	8.02	20.23	6	0.55	−15.72	1	0
71	Stigmasterol	412.70	1	1	7.80	20.23	5	0.55	−15.42	1	0
72	Campesterol	400.69	1	1	7.63	20.23	5	0.55	−15.05	1	0
73	Vitexin	432.38	10	7	0.09	181.05	3	0.17	−4.12	1	1
74	Isovitexin	432.38	10	7	0.09	181.05	3	0.17	−4.12	1	1
75	Secoisolariciresinol diglucoside	686.70	16	10	−2.23	257.68	15	0.17	−2.59	3	2
76	Levulinic acid	116.12	2	1	0.44	54.37	3	0.55	−1.51	0	0
77	Palmitic acid	256.43	1	1	5.55	37.30	14	0.17	−10.03	1	1
78	Oleic acid	282.47	1	1	6.11	37.30	15	0.17	−11.08	1	1
79	Linoleic acid	280.45	1	1	5.88	37.30	14	0.17	−10.77	1	1
80	Capric acid	172.27	1	1	3.21	37.30	8	0.55	−5.98	0	0
81	2‐Decenoic acid	170.25	1	1	2.99	37.30	7	0.55	−5.67	0	0
82	E‐11‐Tetradecenoic acid	226.36	1	1	4.55	37.30	11	0.17	−8.38	0	1
83	Senkyunolide A	192.26	2	0	2.75	26.30	3	0.55	−5.74	0	0
84	Elemicin	208.26	3	0	2.44	27.69	5	0.55	−5.31	0	0
85	Apiol	222.24	4	0	2.16	36.92	4	0.55	−5.08	0	0
86	Dillapiole	222.24	4	0	2.16	36.92	4	0.55	−5.08	0	0
87	Spathulenol	220.36	1	1	3.39	20.23	0	0.55	−7.12	0	0
88	Lutein	568.89	2	2	10.40	40.46	10	0.17	−20.63	2	0
89	Atropine	289.38	4	1	1.93	49.77	4	0.55	−5.41	0	0
90	Hyoscyamine	289.38	4	1	1.93	49.77	4	0.55	−5.41	0	0
91	Scopolamine	303.36	5	1	0.92	62.30	4	0.55	−4.03	0	0
92	Isovaleric acid	102.13	1	1	1.12	37.30	2	0.55	−2.44	0	0
93	1‐Nonadecene	266.51	0	0	7.43	0.00	16	0.17	−12.86	1	1
94	Nonadecane	268.53	0	0	7.66	0.00	16	0.17	−13.21	1	1

**FIGURE 4:**
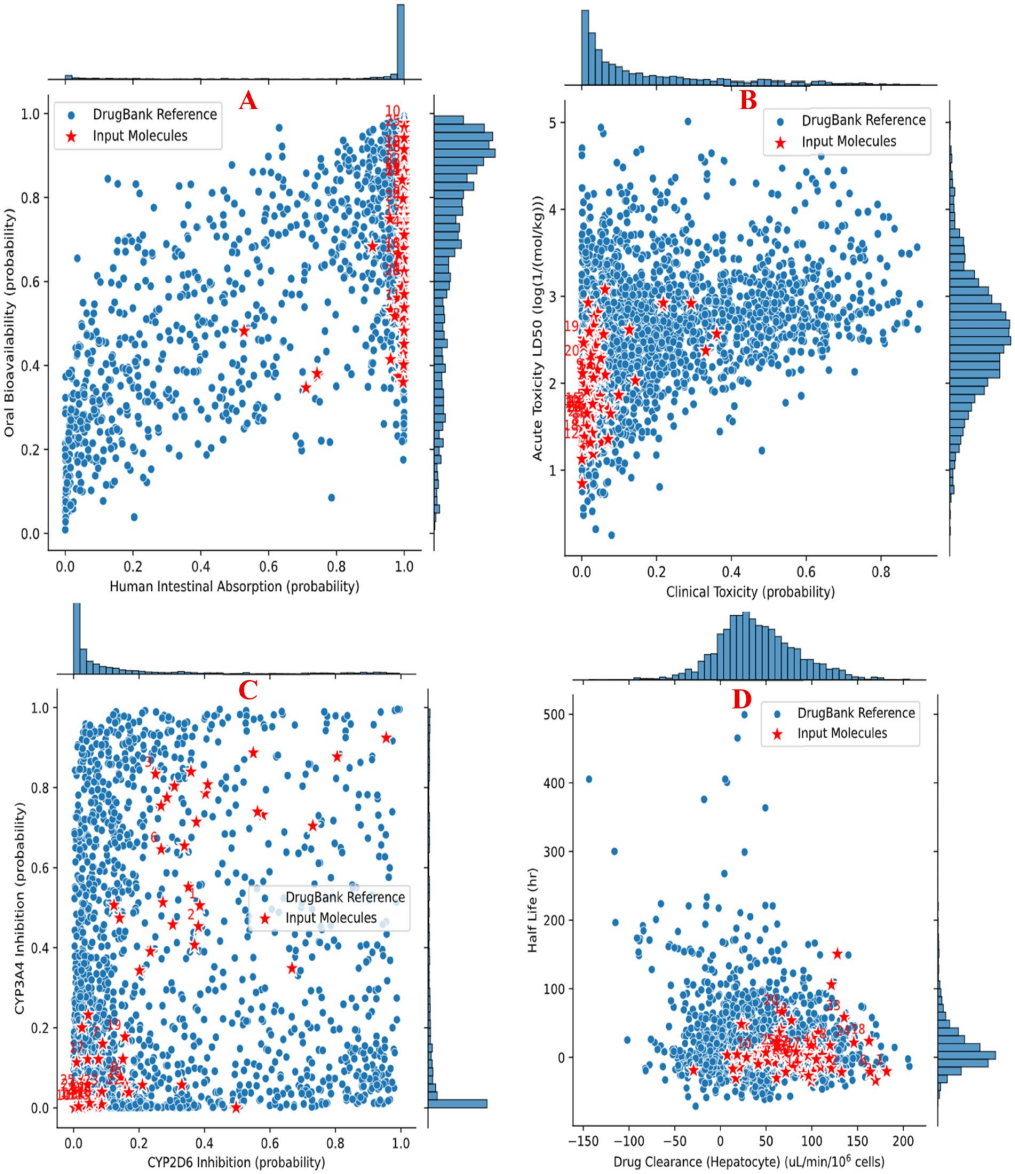
ADMET landscape of the prioritized major phytoconstituents relative to DrugBank. (A) Human intestinal absorption probability versus oral bioavailability probability. (B) Clinical toxicity probability versus acute toxicity (LD50, log scale). (C) Predicted CYP2D6 versus CYP3A4 inhibition probabilities (drug–drug interaction liability). (D) Hepatocyte clearance versus half‐life (pharmacokinetic balance). Blue dots represent the DrugBank reference space, red stars denote the input molecules; marginal histograms show the corresponding univariate distributions.

Figure 4 places the input phytochemicals within the DrugBank reference space using ADMET‐AI predictions. Most compounds fall near regions compatible with drug‐like absorption and manageable toxicity/metabolic profiles, but this does not override compound‐specific limitations identified by SwissADME. In particular, molecules with extreme logS, cLogP, TPSA, or size values were treated as cautionary candidates even when their docking scores were favorable (Fu et al. 2024; Irfan et al. 2024; Mellaoui et al. 2024; Tayebi et al. 2026). The combined interpretation therefore supports prioritizing smaller flavonoids such as baicalein and liquiritigenin for dynamic validation, while reserving highly lipophilic or high‐molecular‐weight hits for future formulation‐focused or mechanistic studies.

### 3.4 Molecular Dynamics

Molecular dynamics simulations were added to address the static nature of docking and to evaluate whether the prioritized ligand‐receptor complexes remained stable under explicit‐solvent, time‐dependent conditions. Liquiritigenin‐ERα and baicalein‐AR were selected because they were among the strongest endocrine docking hits, had biologically relevant receptor associations, and displayed more acceptable Lipinski/Veber profiles than larger or poorly soluble hits such as lutein, procyanidins, and phytosterols. Thus, the MD analysis was used as a second‐level validation step to examine RMSD stability, residue flexibility, global compactness, and persistence of protein‐ligand hydrogen bonding over 200 ns (Bendaoud et al. 2026; Waiba et al. 2025).

**FIGURE 5:**
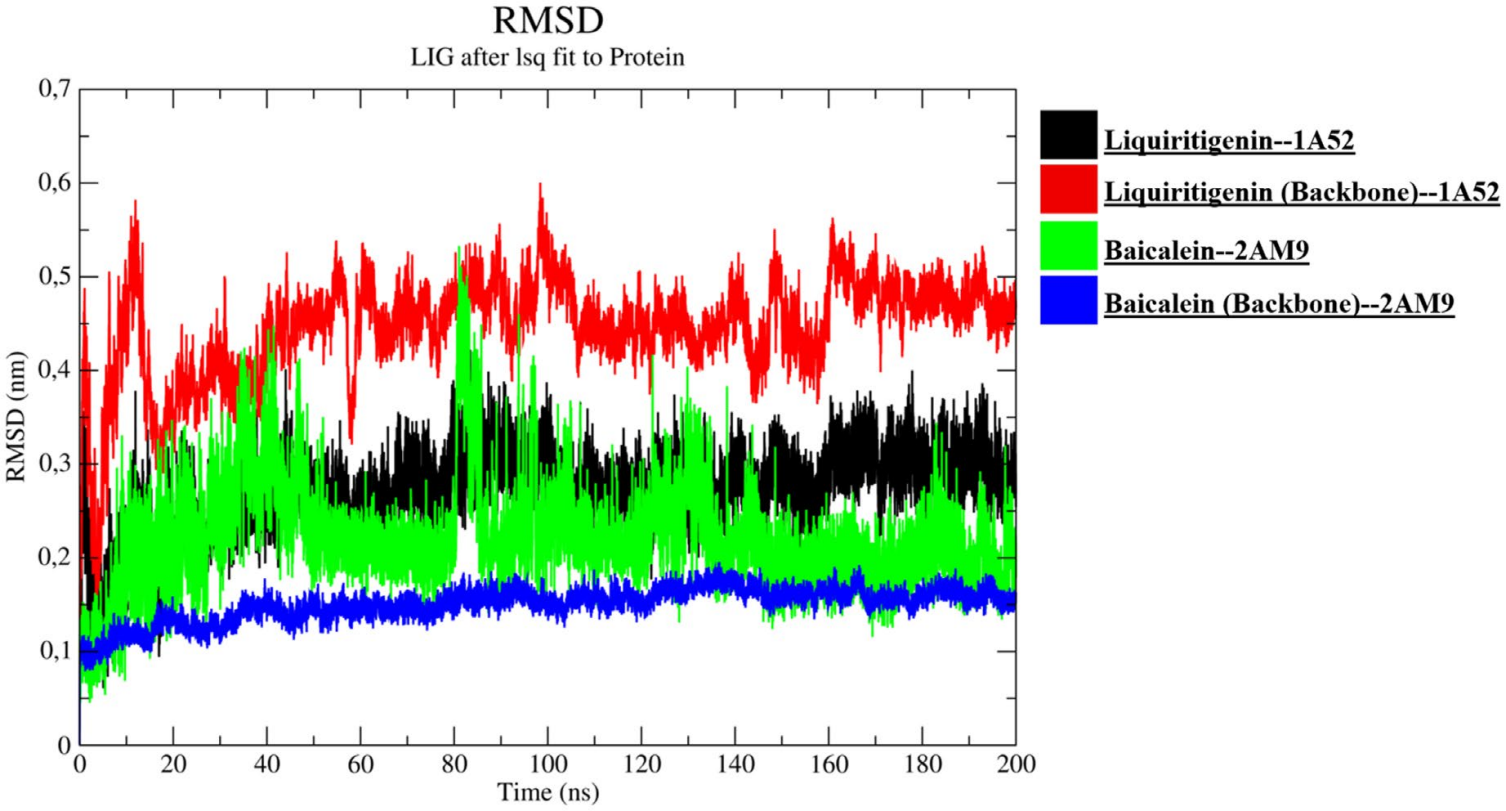
RMSD evolution over 200 ns for the selected ligand‐target systems after least‐squares fitting to the protein backbone. Liquiritigenin‐ERα (1A52) and baicalein‐AR (2 AM9) are shown for both ligand and protein‐backbone trajectories.

The RMSD profiles (Figure 5) show that the AR backbone in the baicalein‐2 AM9 system remained highly stable after early equilibration, generally fluctuating around 0.12–0.18 nm. The ERα backbone in the liquiritigenin‐1A52 system displayed a larger displacement, mostly around 0.40–0.50 nm after equilibration, indicating a more pronounced but still bounded conformational adjustment. The ligand RMSD traces remained mainly within approximately 0.20–0.35 nm, with transient excursions but no sustained drift out of the binding pocket. These patterns suggest that both ligands retained stable receptor‐associated conformations during the 200 ns simulations, with baicalein‐AR showing the more rigid protein framework.

**FIGURE 6:**
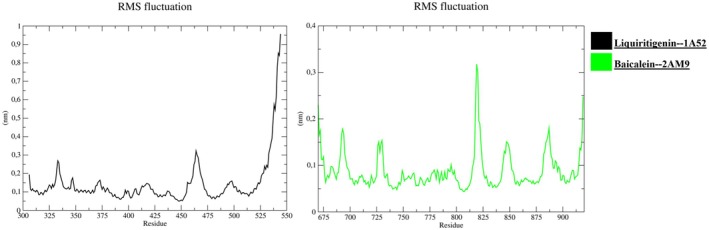
Residue‐wise RMSF profiles of ERα in the liquiritigenin‐1A52 complex and AR in the baicalein‐2 AM9 complex during the 200 ns simulations. Peaks indicate more flexible loop or terminal regions, whereas lower values indicate rigid binding‐domain segments.

The RMSF profiles (Figure 6) further indicate that most binding‐domain residues remained within low‐to‐moderate fluctuation ranges. In the liquiritigenin‐ERα system, the central residues generally fluctuated below approximately 0.15 nm, whereas the largest peak appeared in the terminal region around residues 535–550, a region expected to be more flexible and not necessarily representative of pocket instability. In the baicalein‐AR system, most residues fluctuated near 0.05–0.10 nm, with localized peaks around residues approximately 690, 725, 820, 850, 890, and the C‐terminal end. These localized fluctuations are consistent with mobile loops or terminal segments, while the core binding residues remained comparatively stable.

**FIGURE 7:**
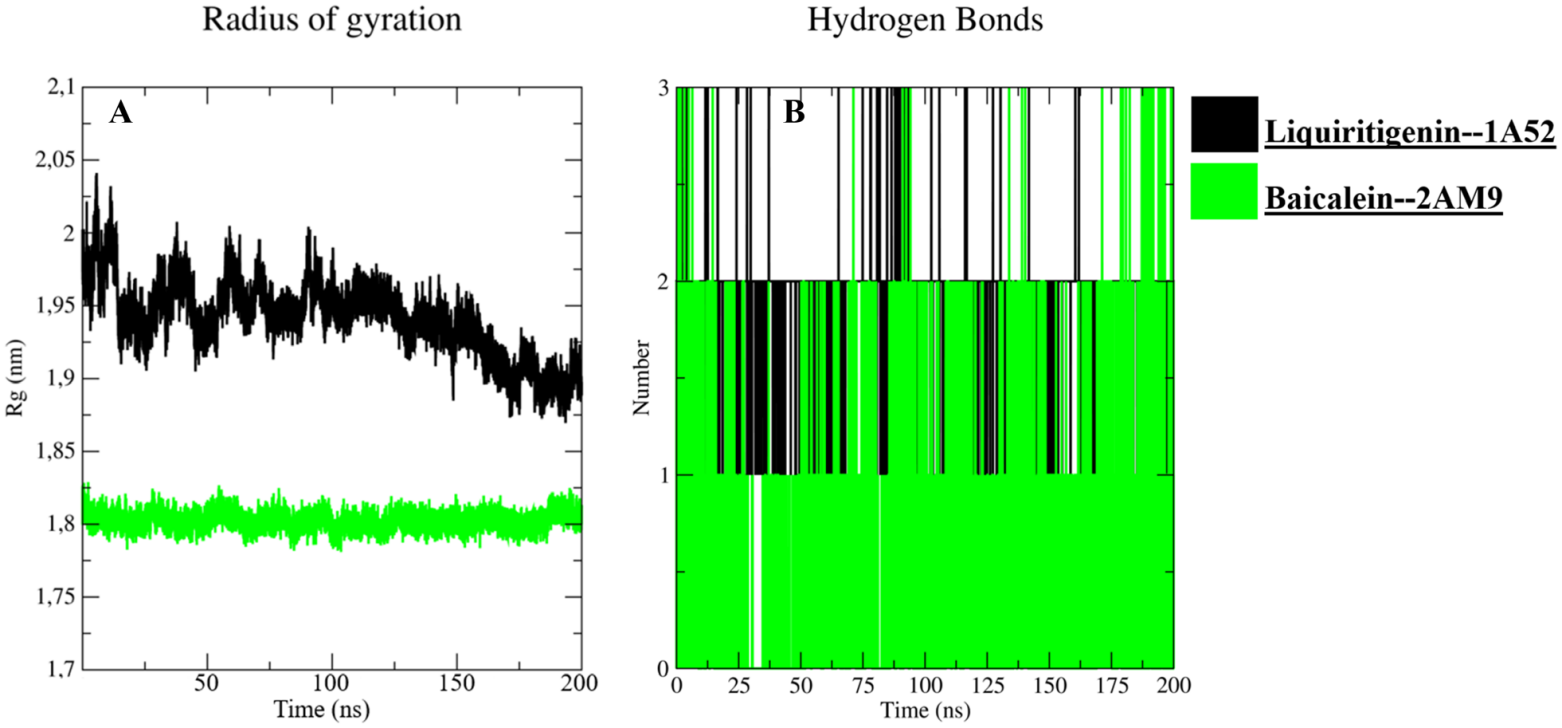
(A) Radius of gyration (Rg) and (B) hydrogen‐bond number for liquiritigenin‐ERα (1A52) and baicalein‐AR (2 AM9) complexes over the 200 ns trajectories.

The radius of gyration and hydrogen‐bond analyses (Figure 7) provide complementary evidence of complex stability. The liquiritigenin‐ERα system showed a gradual Rg decrease from about 1.98 nm toward approximately 1.90 nm, suggesting moderate compaction during the trajectory, whereas baicalein‐AR remained narrowly distributed around 1.80 nm, consistent with a compact and stable receptor fold. Hydrogen bonds were intermittent rather than continuously fixed, fluctuating mainly between 0 and 3 contacts in both systems. This behavior is expected for flavonoid‐receptor interactions in solution, where transient hydrogen bonding is supported by persistent hydrophobic and aromatic contacts. Overall, the MD descriptors support the stability of both prioritized complexes and justify their subsequent MM/GBSA analysis.

### 3.5 MM/GBSA Binding Free Energy

To complement docking and MD, MM/GBSA calculations were performed on 100 evenly distributed snapshots from the equilibrated 175–200 ns trajectory segment. This late‐trajectory window was selected to estimate binding free energy after the main conformational relaxation phase and to reduce bias from early equilibration. Although MM/GBSA remains an approximate end‐point method and does not replace calorimetric or biochemical measurements, it provides useful comparative insight into the energetic stability of the two prioritized complexes (Genheden and Ryde 2015; Basnet et al. 2023).

**FIGURE 8:**
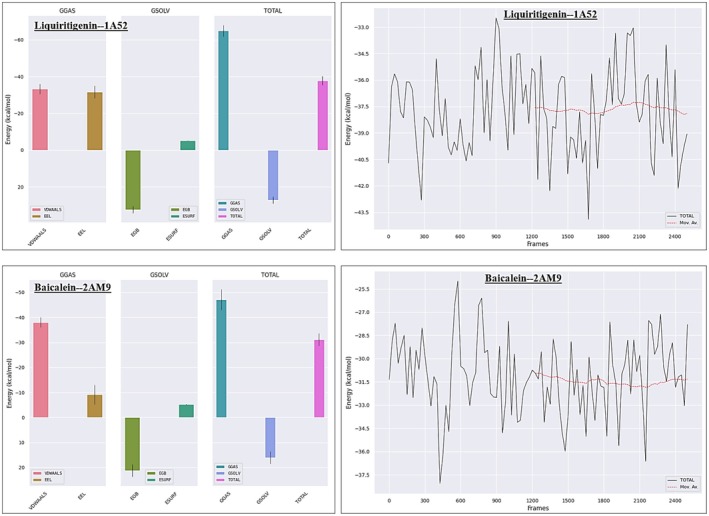
MM/GBSA binding‐free‐energy profiles for liquiritigenin‐ERα (1A52) and baicalein‐AR (2 AM9), calculated from 100 snapshots extracted from the equilibrated 175–200 ns trajectory window. Bar plots show gas‐phase, solvation, and total energetic terms; line plots show frame‐wise total binding energy and moving average.

The MM/GBSA results (Figure 8) indicate favorable predicted binding for both systems. The liquiritigenin‐ERα complex displayed an average total binding energy centered around approximately −37 to −38 kcal/mol, whereas baicalein‐AR was centered around approximately −31 to −32 kcal/mol. In both complexes, the gas‐phase term was favorable and dominated by van der Waals and electrostatic contributions, while the polar solvation term opposed binding, as typically observed for ligand burial in protein pockets. The moving‐average traces remained relatively stable during the 175–200 ns interval, supporting the use of this window for energetic analysis and suggesting that the two complexes did not undergo major late‐trajectory destabilization.

**FIGURE 9:**
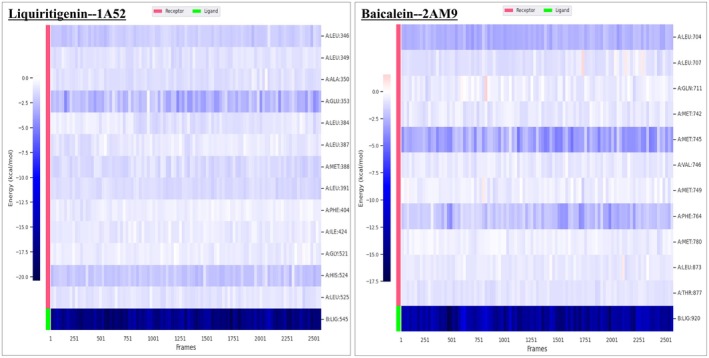
Per‐residue MM/GBSA energy decomposition heatmaps for liquiritigenin‐ERα (1A52) and baicalein‐AR (2 AM9) calculated from 100 snapshots between 175 and 200 ns. Blue regions correspond to favorable residue contributions, whereas white/red regions correspond to weak or unfavorable contributions.

Per‐residue decomposition (Figure 9) identifies the residues that most consistently contributed to complex stabilization. For liquiritigenin‐ERα, favorable contributions were distributed across residues lining the ERα ligand‐binding pocket, including hydrophobic residues such as Leu346, Leu349, Leu384/Leu387, Met388, Leu391, Phe404, Ile424, His524, and Leu525, with Glu353 also contributing to the polar recognition environment. For baicalein‐AR, the most favorable zones involved residues such as Leu704, Met742, Met745, Val746, Met749, Phe764, Met780, Leu873, and Thr877, consistent with the docking interaction map in which Met745 and neighboring hydrophobic/aromatic residues stabilize the flavone scaffold. These data support a binding mechanism in which transient hydrogen bonds act together with persistent hydrophobic and aromatic packing to maintain ligand retention.

Taken together, the MD and MM/GBSA analyses strengthen the prioritization of liquiritigenin and baicalein as computational leads for ERα‐ and AR‐related mechanisms, respectively. However, the findings remain predictive and should be verified experimentally using receptor‐binding or reporter assays, enzymatic/cell‐based models where appropriate, and pharmacokinetic and formulation studies.

## 4 Conclusion

In conclusion, this integrative computational‐phytochemical investigation prioritized Moroccan aphrodisiac phytochemicals with predicted affinity for key targets involved in sexual function and hormonal regulation. Molecular docking identified high‐priority candidates across PDE5, AR, ERα, and aromatase, while ADMET filtering showed that docking strength must be balanced against solubility, size, polarity, lipophilicity, and potential metabolic liabilities. The added 200 ns MD simulations and MM/GBSA calculations further supported the stability and favorable predicted binding energetics of the two ADMET‐compatible endocrine hits, liquiritigenin‐ERα and baicalein‐AR. These results provide a mechanistic and hypothesis‐generating foundation for valorizing Moroccan medicinal plants, but they do not constitute experimental proof of aphrodisiac, receptor‐modulating, or enzyme‐inhibitory activity. Future work should validate the prioritized compounds using enzyme inhibition assays, receptor‐binding or reporter‐cell assays, targeted in vitro models, formulation/solubility testing, and appropriate in vivo studies before any clinical or nutraceutical application is proposed.

## Author Contributions

**Amal Elrherabi:** conceptualization, methodology, writing – original draft, writing – review and editing, validation, visualization, resources, software. **Oussama Khibech:** conceptualization, methodology, writing – original draft, writing – review and editing, data curation, validation, visualization, software, resources. **Mohamed Bouhrim:** methodology, writing – review and editing, validation, visualization. **Mohamed Bnouham:** conceptualization, supervision, project administration. **Abdelaaty A. Shahat:** investigation, funding acquisition. **Joe Miantezila Basilua:** writing – review and editing, software, data curation, formal analysis, validation, visualization. **Abdelouahid Laftouhi:** writing – review and editing. **Abdullah R. Alanzi:** investigation, funding acquisition. **Rashed N. Herqash:** investigation, funding acquisition.

## Funding

This research was funded by the Ongoing Research Funding program (ORF‐RICSP‐2026‐8) at King Saud University, Riyadh, Saudi Arabia.

## Conflicts of Interest

The authors declare no conflicts of interest.

## Data Availability

The SMILES representations of the 94 compounds investigated in this study, together with their compound names and all molecular docking output files, are freely available in the following public GitHub repository: https://github.com/khibech/Moroccan‐Aphrodisiac‐Plants.
